# On the Condition of the Mouth and Teeth during Pregnancy

**Published:** 1875-01

**Authors:** Oakley Coles


### ARTICLE IV.

### *On the Condition of the Mouth and Teeth During Pregnancy.*—Continued.

BY MR. OAKLEY COLES.

In Rigby's "System of Medicine" (1853, p. 63,) under the head of Disorders of Pregnancy, the author says:—"The breath is usually sour, and an acid state of the saliva is indicated by the instantaneous reddening of litmus paper laid upon the tongue." There is yet another mode in which increased flow of saliva and the production of an acid reaction may be produced, and that is by the gratification of the morbid taste that sometimes occurs in pregnant women for acid drinks and sour and unripe fruits.

Salivation may occur, but it is rather rare, and, when present, is "easily distinguished," says Dr: Montgomery (quoted by Dr. Churchill, "Manual of Midwifery," fourth edition, 1860, p. 129,) "from the ptyalism induced by mercury, by the absence of sponginess and soreness of the gums, and of the peculiar fœtor, and by the presence of pregnancy."

Hoppe-Seyler (quoted by Wedl, "Pathology on the Teeth," 1873, p. 336) states that after prolonged fasting, and particularly after continued talking, the saliva may become acid. Both of these conditions for inducing acidity generally obtain in women during the pregnant state.

Wright ("Physiology and Pathology of the Saliva," 1842, quoted by Wedl, *op. cit.*, pp. 355, 356,) has observed that there are certain acids present in various specific diseases. Thus acetic acid is found in the saliva with aphthæ, indigestion, and after the use of acid wines, whilst hydrochloric acid is found in connection with simple gastric disturbances. The same observer has noticed that in facial neuralgia the saliva is alkaline, whilst in rheumatic pains in the face the saliva is acid. As these various conditions may arise in the course of pregnancy, they are of interest in the consideration of the subject before us. Beyond the changes that take place in the saliva we must notice the alteration in the connection of the mucus of the mouth. This, like the saliva, is secreted in greater quantities in most mouths during the earlier months of pregnancy ; and although, in itself, comparatively innocuous, yet from its peculiar physical character and chemical composition (being rich in albuminoid principles) it tends largely to promote an acid fermentation, by combining with the particles of food lodged between the teeth. (Tomes "Dental Surgery," 2nd edition, 1873, pp. 555, 557.)

By fermentation we may have lactic acid formed in the mouth. (Tomes, *op. cit.*, p. 724.

We thus see under certain conditions the saliva itself may become acid in its re-action; that the buccal mucus, by acting upon the decomposing animal tissues lodged around the teeth, may set up an acid fermentation; and lastly, that the mouth is likely to be invaded by acid agents in pyrosis and morning sickness. I can hardly imagine that the alkalinity of the salivary or mucous secretion that can have any injurious effect upon the teeth, although Dr. Garretson ("A System of Oral Surgery," 1873, p. 255) states that in a condition which he describes as oral typh fever, "the patient may be said to labor under the dyscrasia of super-alkaline poisoning, the agent having its point of exhibition most markedly in the mouth."

Whilst Professor Gorgas (Harris's "Principles and practice of Dentistry," 10th edition, 1871, p. 280,) considers that caries "results more frequently from the acid contained in the mucous fluids of the mouth, than * * * from such acids as may be generated by the acetous fermentation of particles of food lodged between the teeth;" but it is certain that from the acidity, however it may arise, we sometimes find the tartar dissolved from the surface of the teeth.

After carefully examining the evidence afforded by published works, and the results of my own observations, with special regard to the action of saliva and mucus during the pregnant state, I cannot but conclude that the great prevalence of caries in women at that period is due in a very large measure to the acid conditions that obtain in the mouth.

It is very remarkable, if simply a coincidence, that the brown variety of caries, which I have found most prevalent in the lower classes, should correspond in its appearance with caries produced artificially out of the mouth by means of lactic and butyric acid.

On the influence of lactic acid, Wedl (*op. cit.*, pp. 418, 419,) mentions that organic chemistry teaches us that it occurs frequently in partially decomposed animal fluids, and Schmidt has demonstrated the presence of this acid in the fluid obtained from long bones affected with osteomalacia; the analogue of which condition I have endeavored to show as occurring in some of the teeth during pregnancy. Fischer has also obtained butyric, valerianic, and formic acids from strongly alkaline or acid pus, and Wedl suggests "whether these may not occur as products of the decomposition of the puriform secretions of the gums."

Although in the majority of cases we find increased flow of saliva and mucus, in other instances the amount seems normal; and in these cases it is that we get the white caries and softening, with fatty degeneration, the gums being anæmic and receding, but still attached to the higher part of the necks of the teeth. Decay is not so rapid under these conditions, and seems to indicate impaired nutrition rather than altered secretions as a cause, strengthening negatively the views put forward as to the influence of increased and acidulated fluids in the mouth.

But even here we must not overlook the effects of localized acidity such as occurs around the margins of the gums, as pointed out by Tomes (*op. cit.*, p. 555,) and we must bear in mind that the symptoms which I have described, such as softening and disintegration, with white caries, may arise from this cause, either alone or in combination with impaired nutrition; for it would, I think, be exceedingly unsafe to declare absolutely the specific cause for a certain condition of the teeth during pregnancy, when there are so many factors operating, the proper value of which it is almost impossible to take into account.

In an earlier part of my paper I referred to the decay that is sometimes seen on the palatine cusps of the bicuspid and molar teeth, and mentioned that this class of caries was interesting from the peculiarity of its situation. There are I think, two explanations that may be offered for this fact (beyond, of course, the consideration of special structural defect;) one is that the inner cusps of the bicuspids and molars are those against which fluid would be most forcibly ejected in vomiting, and partially so in pyrosis; and further that the tongue, when at rest, very often lies in contact with these teeth, and so the secretion of acid mucus from the side of the tongue would in time operate injuriously by dissolving rhe enamel.

Before leaving the subject of saliva, it may be well to state that we must not attach an undue importance to its acid condition as a cause of caries during pregnancy, since in healthy males there is occasionally an acid reaction in the intervals of digestion, and in certain acute diseases the saliva is very commonly acid; in fact, it is almost the rule to find the saliva giving an acid reaction under such circumstances.

4. *The Neuralgia of Pregnancy.*—When pain arises in the face or in the regions of one or more teeth during pregnancy, without the presence of caries, we may reasonably ascribe it to a neuralgic origin. Whilst, further, neuralgia thus occurring may have no relation to the teeth, but be entirely dependent on either impaired or perverted nutrition. (Anstie.)

It is therefore not singular that we should find it associated with an anæmic condition of the system and a wasting, rather than carious, condition of the teeth.

This view is clearly set forth in Aitkin's "System of Medicine" (1868, vol. ii. pp. 508-510,) where the author states that facial neuralgia occurs, in the great majority of cases, in patients of an anæmic condition, occurring more frequently before thirty than afterwards, and especially in those whose menstruation is irregular either as to time or quantity.

Ramsbotham ("Obstetric Medicine and Surgery," 4th edition, 1865, p. 646,) writing of the annoyances connected with the pregnant state, says, "Toothache and facial neuralgia are the most common; violent pain is referred to one tooth, or perhaps to the whole of one side of the jaw, without the presence of caries." And again, Murphy (" Manual of Midwifery," and edition, 1862, pp. 53, 92,) writing on toothache, says, " Pain in the ear and face sometimes causes distress, but the most frequent source of misery is toothache. Some caution is necessary when such a symptom presents itself, especially if there be a decayed tooth. The patient may fly for relief to the usnal remedy—extraction. This, however, does not afford the customary benefit. The pain may be removed for a short time, but it soon attacks another tooth, and thus renders extraction useless.

In Tome's " Dental Surgery," p. 367, it is stated that " the conditions which seem more often to predispose to neuralgia are the exhaustion of over-work; women are also subject to neuralgia, as opposed to toothache, in the early months of pregnancy."

It would enlarge too much the present paper to give all the extracts bearing upon the subject of neuralgia during pregnancy, that are to be found in the various works on midwifery and nervous diseases. The condition is one that has been fully recognized, and needs, I think, no further evidence in support of the fact of its occurrence.

In most cases the neuralgia is more severe in the first pregnancy than in the subsequent ones; and this remark applies also to toothache, for, although the caries may advance with successive pregnancies, the pain is much more severe in the early pregnancies than afterwards.

5. There are other changes that take place in pregnancy that demand some attention.

" The blood has a rather lower specific gravity than the average, from the deficiency of blood corpuscles. The quantity of white corpuscles and of fibrine, on the other hand, is increased. (Kirke's " Physiology," 5th edition, p. 94.)

And further there is an almost unexplained liability in pregnant and puerperal women to plugging of the veins (thrombosis.) It is not impossible that this fact may in some measure account for the impaired nutrition, from which the teeth certainly in some suffer.

The dropping out of the teeth is, there can be very little doubt, due to an arrested nutrition, and I think has its analogue in the transverse markings of the nails, met with after severe and exhausting diseases

Wedl, in the "Atlas of Dental Pathology" (plate 5. fig. 50,) shows a case of thrombosiis occurring in the pulp of a tooth, and mentions that "it sometimes is seen in the pulps of old permanent and temporary teeth that are in a state of resorption." Impaired nutrition, manifested locally, may however, be due to a perversion of the nutritive elements, or rather the direction of nutrition into a new channel, in order to supply the wants of the growing fœtus. Whether there is resorption of the earthy salts of the tooth, to supply the fœtus with earthy salts, is a question that is open to doubt, but there certainly seems reason for believing that the deposition of the calcareous matter may not take place, and thus produce a condition equivalent to that in which resorption is supposed to occur.

6. *The Remedial Agents useful during Pregnancy.*—In all affections of the mouth and teeth that may come under the notice of the dental surgeon, it is well to see that the general health is being kept up to the highest state of excellence consistent with the pregnant state.

The diet should be simple, nourishing and non-stimulating,—as nearly approaching that of the ordinary regime as possible. But above all things, I believe the use of oatmeal in any form as an article of diet to be of the greatest value to both the mother and child. It may be taken either as oat-cake or in the form of porridge with milk or cream.

The taste for unusual dishes should be controlled, and the longing for sour fruit and acid drinks checked, though the derangement of the digestion, of which the craving is but a symptom, must be fully recognized, and proper steps be taken to rectify it. Where there is increased acid secretion, either from the mouth or stomach, acids should be administered internally, every precaution being taken that their influence is limited to the stomach, and avoiding as far as possible their action on the oral and buccal cavities. For this purpose, it is extremely important that the acids employed be administered—within half an hour of taking a meal—by means of a tube, the mouth being immediately rinsed out, either with water or with an alkaline gargle. By this means we secure all the advantages of the acid without any of its local ill effects. Either nitric or hydrochloric acid, or the two combined, may be employed for this purpose. Ten drops of dilute nitric acid with bitter infusion, such as chiretta or calumba, with half a drachm or a drachm of tincture of orange peel, is a convenient method of administering the acid.

The carious cavities in the teeth should be filled up when it is possible, in order to allay local irritation and pain; and for this purpose I always found Hill's stopping or Jacob's gutta-percha the best agents. They are bad conductors and non-irritating,—both qualities of great value in a stopping that is applied at such a time.

When the teeth are the subjects of soft caries, or softening or fatty degeneration of the entire tooth, accompanied with great sensitiveness, I have found collodion of the greatest value. The tooth must first be wiped over with cotton wool saturated with alcohol, to remove the greasy surface and absorb any moisture. The collodion may then be painted over the whole of the tooth that appears through the gum, and affords a most efficient yet simple protection against thermal change and irritating substances or liquids. This may be done thrice daily, but as a rule, twice a day will be found to be sufficiently frequent.

Dr. Kock, of Chicago ("Missouri Dental Journal," April, 1873,) recommends covering the teeth and gums with prepared chalk, for allaying the sensitiveness of the teeth, where there is no caries.

When the teeth appear to be breaking down from the severe strain which the pregnancy puts upon all the resources of the patient, cod-liver oil in small and gradually increasing doses will be found beneficial. As a tooth-powder, precipitated chalk I have found the best, since it not only neutralizes the acidity, but tends to check the flow of saliva if it be an excess.

I have also found charcoal used at night, with plenty of water and a soft badger-hair tooth brush, most serviceable as an antiseptic agent for the teeth and gums, and may also be used as a wash for the mouth before going to bed; thus checking decomposition and acid fermentation from the particles of food that have accumulated between the teeth.

Too much importance cannot be attached to the careful use of the tooth brush before retiring to rest, as it is when the mouth is at rest that a good deal of mischief is done to the teeth. Quinine and chalk as a dentifrice (ʒ j. to ℥ ij.) are also good in some cases in allaying pain of a neuralgic character, and may be ordered with great advantage.

In periostitis, the usual remedies, such as iodine tincture and carbolic acid seem the most valuable; whilst both of these agents diluted may be used as a stimulating application to the gums where there are apparently impaired nutritive processes.

The carbolic acid is also very good where there is a deficient secretion of saliva, such as sometimes occurs; the most convenient form is the following:—

| | | |
|---|---|---|
| ℞. | Acid. carbolic. | gr. xij. |
| | Glycerine. | ʒ ss. |
| | Aquæ Rosæ | ℥ j. |

In those cases in which a purulent discharge arises from around necks of the teeth, the chloride of zinc (gr. iij. to ℥ j.) is useful; it must be applied well with a brush, so as to thoroughly enter between the tooth and gum.

Chlorate of potash and bromide of potassium may also be used sometimes with advantage in certain conditions of the gums and mucous membrane; chlorate of potash being valuable on account of its healing and antiseptic properties and bromide of potassium or its reputed sedative and anæsthetic action.

The treatment of neuralgia must vary according to the cause of the disease and the condition of the patient; as a rule, local stimulation is beneficial, by means of either a mustard plaster or chloroform applied with liniment ( ℥j. to ℥vij.); sal volatile, hot tea, large doses of quinine, small doses of alcohol, and light and nourishing food, so as to restore the impaired condition of the nervous system. (Aitkin.)

The remedy that I have however, found the most useful is the syrup of the phosphate of iron (Parrish's Chemical Food,)—a teaspoonful twice daily, ten minutes after each meals. This, in my own experience, I have seen give greater relief than many of the agents I have mentioned. Under all circumstances, the treatment must be as gentle as is consistent with efficiency, and all shock saved the already overwrought nervous system of the patient.

Tooth-extraction for odontalgia or neuralgia must never be undertaken without a full recognition of the risk incurred by the mother and child, and the responsibility attaching to the operator; and when absolutely necessary, is, probably, less injurious if performed under the influence of an anæsthetic, such as nitrous oxide or ether or the two combined.—*Transactions of Odontological Society of Great Britain.*

---

